# Filamentous Fungus *Aspergillus oryzae* for Food: From Submerged Cultivation to Fungal Burgers and Their Sensory Evaluation—A Pilot Study

**DOI:** 10.3390/foods10112774

**Published:** 2021-11-11

**Authors:** Neda Rousta, Coralie Hellwig, Steven Wainaina, Lukitawesa Lukitawesa, Swarnima Agnihotri, Kamran Rousta, Mohammad J. Taherzadeh

**Affiliations:** Swedish Centre for Resource Recovery, University of Borås, SE-501 90 Borås, Sweden; coralie.hellwig@hb.se (C.H.); steven.wainaina@hb.se (S.W.); lukitawesa.lukitawesa@hb.se (L.L.); swarnima.agnihotri@hb.se (S.A.); kamran.rousta@hb.se (K.R.); mohammad.taherzadeh@hb.se (M.J.T.)

**Keywords:** food security, sustainability, circular economy, functional food, myco-food, meat alternative

## Abstract

New food sources are explored to provide food security in sustainable ways. The submerged fermentation of edible filamentous fungi is a promising strategy to provide nutritious and affordable food that is expected to have a low environmental impact. The aim of the current study was to assess the novel use of *Aspergillus oryzae* cultivated in submerged fermentation on oat flour as a source for food products that do not undergo secondary fermentation or significant downstream processing. The fungus was cultivated in a pilot-scale airlift bioreactor, and the biomass concentration and protein content of the biomass were assessed. A tasting with an untrained panel assessed consumer preferences regarding the taste and texture of minimally processed vegetarian and vegan burger patties made from the biomass, and how the patties fared against established meat-alternative-based patties. The cultivation of *Aspergillus oryzae* resulted in a yield of 6 g/L dry biomass with a protein content of 37% on a dry weight basis. The taste and texture of the minimally processed fungal burger patties were to the liking of some participants. This was also reflected in diverse feedback provided by the participants. The cultivation of the fungus on oat flour and its utilization in developing burger patties shows its promising potential for the production of nutritious food. The applications of the fungus can be further developed by exploring other favorable ways to texture and season this relatively new functional food source to the preferences of consumers.

## 1. Introduction

Efforts are currently being directed to the exploration of new food sources given the increase in the global population and the demand to secure nutritious, sustainable and affordable food for everyone. Once such food sources are found, encouraging the consumption of food products that are expected to be socially, financially and environmentally sustainable can make an important contribution to achieving sustainability targets [1] such as the Paris Climate Accord or the United Nations Sustainable Development Goals (i.e., zero hunger, good health and well-being, responsible consumption and production, and climate action). 

Meat has long been a major source of human diet [2], but it has also been associated with some major drawbacks. Some of these shortcomings are connected to the high demand for land and water, a potential loss of diversity and the high emission level of greenhouse gases in the meat supply chain [3]. Moreover, the consumption of meat can have negative effects on human health. For example, a high intake of red meat increases low-density lipoprotein (LDL), leading to coronary artery disease [4]. On the other hand, inadequate protein consumption can result in muscle waning, edema, and a severe protein deficiency can lead to protein-energy malnutrition [5,6,7]. Accordingly, food products that aim to replace meat should aim to provide the same nutritional benefits as meat. Plant-based diets have increasingly been perceived as more suitable compared to meat-based counterparts [8]. However, not all plants provide adequate amounts of essential amino acids [9]. Plant-based protein sources can also contain antinutritional compounds such as phytate, that decrease the bioavailability of plant-based nutrition in the human digestive system [10].

Edible filamentous fungi produce a variety of enzymes that allow them to grow on different types of substrates and produce a fungal biomass that is rich in nutrients. However, the potential of edible filamentous fungi as new sources of food and as meat alternatives has yet to be fully explored. Filamentous fungi are not only promising substitutes to meat in terms of nutrition and affordability but are also expected to have a lower environmental impact than meat [11,12,13]. Indeed, filamentous fungi can improve the nutritive values of substrates as well as improve bioaccessibility and the absorption of nutrition in the human digestive system by synthesizing vitamins, decreasing antinutritional compounds and converting amino acids and lipids [14,15,16]. The fungal biomass derived through fermentation not only meets basic nutritional requirements but in addition to the above-mentioned nutrients, it is also a valuable source of bioactive compounds such as antioxidants, minerals, polyunsaturated fatty acids and fibers (e.g., β-glucans) which enhance health benefits and reduce the risk of developing certain diseases [17,18]. Because of the improved nutritional profile, food produced through fungal-fermentation is considered functional [19,20]. Filamentous fungi have traditionally been used in the production of fermented food for human consumption. *Neurospora intermedia* and *Rhizopus oligosporus* are two commercially exploited fungi that are used in food production such as oncom and tempeh [21].

Solid-state fermentation (SSF) is applied in the production of many traditional fermented food products. Although SSF is associated with low water and energy consumption [22], scaling up SSF-based production is considered difficult and is thus mainly limited to small-scale production of fermented food [22]. Submerged fermentation (SmF) has been used on a more minor scale than SSF to produce fermented food. However, the industrial cultivation of filamentous fungi to produce enzymes, organic acids and bioactive compounds is commonly performed in SmF. The benefits of using SmF include the availability of a wide range of reactor designs, an easily scalable production process, and extensive documented research gained through industrial applications [23]. Additionally, SmF provides opportunities to co-produce bioactive compounds in a higher concentration than possible to date in SSF. An example of this includes the production of L-carnitine by *Aspergillus oryzae (A. oryzae)* in a semi-synthetic medium in a submerged cultivation [24]. Examples of food products made from filamentous fungal biomass (*Fusarium venenatum*) through SmF are products by Quorn™ [25].

*A. oryzae* is generally regarded as safe (GRAS) [26] and the use of its biomass for food production is not considered to be novel within the EU (e.g., [27]) The fungus or ‘koji mold’, has been cultivated in SSF and used for centuries in the production of indigenous Asian foods such as rice wine (sake), soy sauce (shoyu), soybean paste (miso), and distilled spirits (shochu) [28]. *A. oryzae* fermented biomass is thus commonly used as a substrate for secondary fermentation [29] but empirical research has yet to assess the cultivation of fungal biomass from *A. oryzae* for direct use in food production. 

*A. oryzae* can be cultivated using a variety of substrates, which include residues from food processing facilities such as thin stillage [30], fish processing wastewater [31], pea-processing byproduct [32], and vinasse [33]. Even though the utilization of the mentioned substrates can contribute to circularity, their utilization for producing fungal biomass intended for direct human consumption might be challenging. This is due to the uncertainties regarding the quality of the substrate source that can be subjected to legal and social criticism [34]. On the other hand, utilizing substrates that are already in the human food market, such as oat flour, satisfies the safety requirements for producing a fermented food product using *A. oryzae*. Furthermore, an oat-based medium contains the necessary nutrients for the cultivation of *A. oryzae* in SmF systems and thus eliminates the need for nutrient supplementation. The use of oat in human diet has also been associated with some health benefits [35] which make it attractive to explore its use in scaled-up fermentation processes.

It is constructive to combine technical aspects and peoples’ perceptions in a study regarding new foods, such as submerged cultivated *A. oryzae*. This is because it is important to assess how sensory characteristics are experienced when aiming to encourage individuals to choose a new product or ingredient over conventional ones [36]. This is especially the case for new food products or ingredients, such as *A. oryzae*, with which people are unlikely to be familiar [37]. 

There are also other aspects that are important to consider when developing new food products and ingredients. Attitudes towards chemicals in food products, for example, lead many people to choose natural foods over food items that contain extensively processed or synthetic ingredients [38]. Simultaneously, minimally processed food is one of the major growth segments in food retail [39]. Minimal processing in the context of food describes techniques in which food items are processed in ways that result in the least change to their inherent characteristics [40]. During the development of new food products or food made with new ingredients, it would thus seem reasonable to assess sensory evaluations when products are minimally processed. This is because this allows for the exploration of the potential of minimal processing methods for the specific product or ingredient. The outcome of such evaluations can constructively assist in the further development of the food product. However, to the authors’ knowledge, there are currently no studies that explore the SmF cultivation of *A. oryzae*, a protein analysis of the biomass, food production using the derived biomass using minimal processing, and a sensory evaluation that assesses how taste and texture of food made with minimally processed *A. oryzae* biomass is received. 

In light of the above, the current study aimed to assess the novel use of (i) submerged fermented *A. oryzae* as a source for food products that do not undergo secondary fermentation, and (ii) to explore minimal processing of the biomass in the production of food products. The objectives were (i) to assess the submerged cultivation of *A. oryzae* on oat flour; (ii) to assess the fungal biomass concentration and protein content of the biomass; and (iii) to assess how the taste and texture of minimally processed burger patties made from the biomass are received.

## 2. Materials and Methods

To achieve the objectives of the current study, the project was conducted through multiple stages. The substrate was prepared first, followed by fungal cultivation, fungal-burger preparation and a sensory evaluation. 

### 2.1. Fungal Biomass Production

#### 2.1.1. Substrate Preparation

Oat flour from AXA (Lantmännen Cerealia, Malmö, Sweden) was used as the substrate. The oat flour was kept in a dry place at room temperature until use. The appropriate amount of substrate was first determined among four selected concentrations, i.e., 30, 40, 50 and 60 g/L, according to the biomass yield and the residual starch in the medium after fermentation. The impact of temperature on the viscosity of oat flour–water mixture was then examined to establish the ideal mixing condition. The specified concentration of oat flour was mixed in 100 mL of varying water temperatures including cold water (22 °C), 50, 60, 70, 80, and 90 °C. Then, the samples were cooled down and the suspensions were equilibrated to room temperature (22 ± 1 °C) before their viscosity measurement. The displayed viscosity after 30 s of measurement was taken as the respective value for each specimen. Each sample was measured three times. Prior to feeding the 26 L bioreactor, 600 g of oat flour was incrementally added to 20 L of water and evenly mixed with a hand-held mixer. Feeding of the 1200 L bioreactor was performed in a similar manner and the substrate concentration was set as 26 L of the bioreactor.

#### 2.1.2. Fungal Cultivation and Scale Up

The production of fungal biomass was performed in three steps. First, the fungal spores were propagated from an existing culture. The spores were then used to prepare a preculture in 1 L shake flasks which served as seeding for a 26 L reactor. Finally, the biomass from the 26 L reactor was used as seeding for the pilot 1200 L air lift reactor.

*A. oryzae* var. *oryzae* CBS 819.72 (Centraalbureau voor Schimmel-cultures, Utrecht, The Netherlands) spores were produced on petri dishes containing a solid PDA (Potato Dextrose Agar) medium with the composition of (in g/L) glucose of 20, potato infusion 4 and agar 15, for 3–5 days at 30 °C. The spore suspension was made via the addition of 20 mL of sterile distilled water to each agar plate with *A. oryzae*. Thereafter, the 20 mL spore suspension was added to 2 L flasks containing 20 g/L oat flour and 10 g/L sucrose for 24 h to provide 1 L pre-inoculum. The spore concentration was 9.6 × 10^7^ spores/mL.

The pre-inoculum was fed thereafter to a 26 L bubble column bioreactor (Bioengineering, Zürich, Switzerland) containing 20 g/L oat flour, 10 g/L sucrose and 100 mL oil. The bioreactor was empty in-situ sterilized by injection of steam at 130 °C for 20 min, while an oat flour mixture was sterilized at 121 °C for 20 min in an autoclave (Systec, Linden, Germany). The aeration rate was 0.5 vvm (volume of air per volume of liquid per minute) and the cultivation was performed at 35 °C without a pH adjustment. 

**Figure 1:**
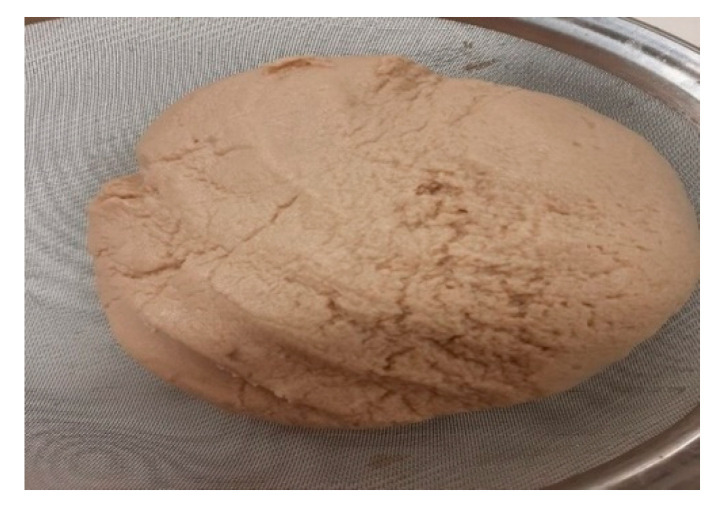
*A. oryzae* biomass after 48 hours of cultivation.

**Figure 2:**
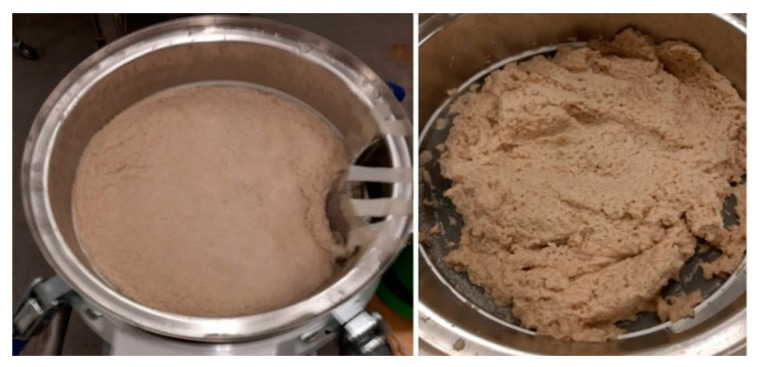
*A. oryzae* biomass during and after sieving using a vibration screen.

**Figure 3:**
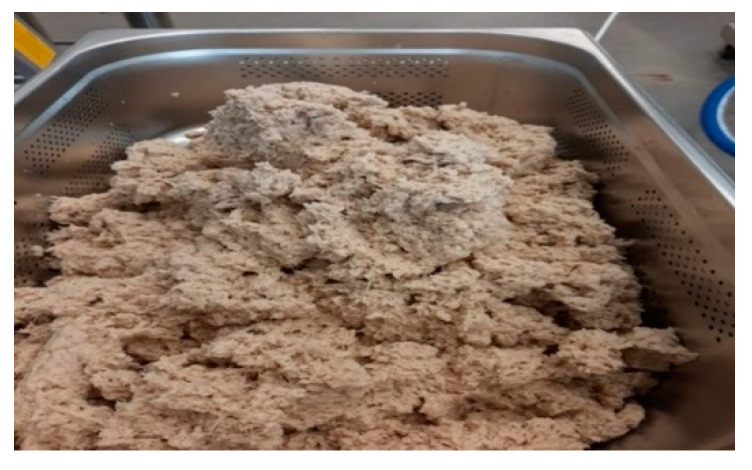
*A. oryzae* biomass after excess liquid removal.

After 24 h of cultivation, 20 L of the biomass suspension was extracted from the 26 L bioreactor and used as seeding for the 1200 L airlift bioreactor (Knislinge Mekaniska Verkstad AB, Kristianstad, Sweden). Before inoculation, the 1200 L bioreactor was sterilized in two cycles; the first cycle involved empty in-situ sterilization through the injection of steam at 121 °C for 20 min while the second cycle involved sterilization with the substrate (30 g/L oat flour mixture) by the injection of steam at 121 °C for 20 min. Prior to inoculation, 1.5 L of cooking oil and 100 mL of antifoam were added to the bioreactor. The aeration rate was 0.45 vvm and cultivation was performed at 35 °C without a pH adjustment. The initial pH was 6.5 whereas at harvest it was 3.8. During cultivation, 100 mL of sample was withdrawn from the bioreactor every six hours and the pH value was recorded. The biomass was recovered from the sample using a stainless-steel kitchen sieve (1 mm^2^ pore area) and weighed via analytical balance to monitor the growth pattern. The biomass was harvested after 48 h of cultivation (Figure 1). The suspension from the bioreactor was sieved using a vibration screen (Russell Compact Sieve^®^, Russell Finex Ltd., Feltham, UK) to remove the liquid fraction (Figure 2). The biomass was then pressed using a 12 L juice press (Bauhaus, Belp, Switzerland) (Figure 3) and stored in a freezer until use. Prior to its analysis, the fungal biomass was freeze-dried at 0.05 bar and −50 °C to a constant weight and pulverized using a ball mill (Retsch MM 400, Haan, Germany) for periods of 25 s at a frequency of 30 Hz.

#### 2.1.3. Analytical Methods

The crude protein content of biomass was measured according to the kjeldahl method using an InKjel P digester and a behrotest S1 distiller (Behr Labor-Technik, Düsseldorf, Germany). Initially, 20 mL of 98% H_2_SO_4_, KT1 and antifoam tablets (Thompson & Capper Ltd., Runcorn, UK), were added to a 0.5 ± 0.00 g dried and pulverized biomass followed by 100 min digestion. In the second step, distillation vapor was collected in 50 mL of 4% H_3_BO_4_. Lastly, titration was carried out with 0.1 M HCl until it reached a pH of 4.6. A factor of 6.25 was used for the conversion of nitrogen-to-protein. The total starch of the liquid fraction was determined according to the enzymatic method AOAC 996.11, using the Total Starch Assay Kit (Megazyme, Wicklow, Ireland). The viscosity of the oat flour–water mixtures were measured with a sinewave vibro-viscometer (SV-10, A&D, Tokyo, Japan).

#### 2.1.4. Statistical Analysis

The shake flasks experiments were carried out in duplicate, and a statistical analysis of the data was performed using MINITAB^®^ 17 (Minitab Ltd., Coventry, UK). The error bars and intervals reported in the text, tables, and graphs represent two instances of the standard deviation. The analysis of variance (ANOVA) was carried out using general linear models, such as the Tukey test.

### 2.2. Sensory Evaluation 

To assist in the development of food products from submerged cultivated *A. oryzae* early on, a tasting was conducted, with the aim to assess how the biomass is perceived when it is minimally processed. This was performed to assess whether minimal processing is adequate for submerged cultivated *A. oryzae* biomass, and to ensure that further developments will appeal to people the products may later cater to. 

The number of participants was expected to be small due to the COVID-19 pandemic. The sensory analysis, however, was conducted to assess data that can provide a general idea of how individuals perceive food they are unfamiliar with, an objective for which a smaller numbers of participants can be sufficient [41,42].

#### 2.2.1. Sample Preparation

**Figure 4:**
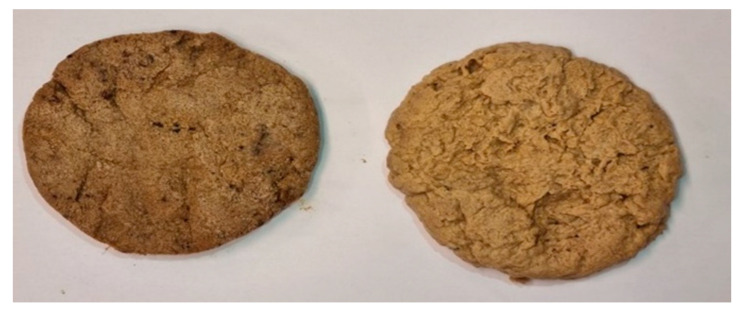
Vegetarian (**left**) and vegan (**right**) burger patties before frying.

**Figure 5:**
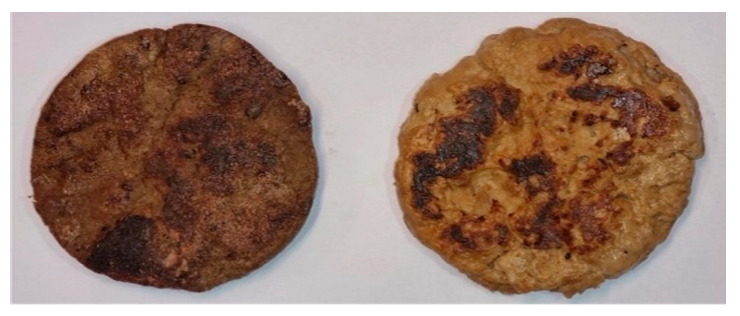
Vegetarian (**left**) and vegan (**right**) burger patties after frying.

Burger patties were produced from the derived biomass because it was assumed that most participants would be familiar with the product if not the fungus used as the main ingredient. This was considered given that unfamiliarity with food can influence a person’s sensory evaluation and lead to low ratings [43]. To prepare the vegan and vegetarian fungi burger patties, 1 kg biomass for each burger option was thawed and then rinsed in a 12 L juice press (Bauhaus, Belp, Switzerland) by first adding water to the biomass and then pressing the water out. Excess liquid was squeezed out once more before the biomass was processed into burger patties. For the vegan burger patties, 200 g of pregelatinized starch as well as a mixture of cornstarch, tapioca starch and Easy Binder™ (Special Ingredients Ltd., Chesterfield, UK) were mixed with the biomass (20% of the total mass). To make the vegetarian version of the fungi-burger patties, 400 mL egg whites were used instead of the starches and binders in the vegan version. Both versions were seasoned with salt, spices and herbs. The burger patties weighed 130 g each, had a diameter of 9 cm and were 1.5–2 cm thick (Figure 4 and Figure 5). To provide participants with a reference point, two other patties that are available on the Swedish market, i.e., Beyond (Beyond Meat^®^, El Segundo, CA, USA) and Quorn (Quorn^®^, Loughborough, UK) were also prepared. Beyond is a plant-based product (derived from peas) while the Quorn burger patty is a fungi-based product derived from *Fusarium venenatum*. All burger patties (vegan and vegetarian (Picture 2) as well as Beyond and Quorn) were fried on medium heat for 2–3 min on each side using rapeseed oil to grease the pans.

#### 2.2.2. Participant Selection

Participants were approached at an event at the University of Borås, Sweden. The event presented an opportunity to approach a group of people of different ages and genders during the COVID-19 pandemic. Among the participants were staff members and students as well as their family members and peers. Participants were asked to read an information sheet about the study and were then asked to sign their consent before participating in the tasting. Participants were also informed that they should not participate in the study if they were aware of any of the following conditions: allergies towards fungi, a compromised immune system, if they were prone to strong allergic reactions, lung issues (e.g., asthma), or digestive sensitivities, such as an easily upset stomach. Ethical approval for this study was obtained from the Swedish Ethical Review Board. 

#### 2.2.3. Data Collection

Data were collected using an adapted version of the descriptive analysis for sensory evaluation. Using this technique, data were assessed, assisting with the identification and description of characteristic attributes as well as the quantification of the perceived intensity of sensory characteristics of food products [44]. Moreover, this technique also aids in the assessment of potential consumer preferences and the ways in which products differ from one another in terms of sensory characteristics [45].

The participants were informed about the identity of the samples both verbally and in writing. They were also informed that the vegan and vegetarian fungi-burger patties were made from fungi that were cultivated and processed in the university’s lab. Because information provision about food products may influence sensory experiences [46], further information about the fungi-based burgers was not provided unless participants inquired. The questionnaire used in the present tasting was specifically tailored to the aims of this study per Lundgren [47]. The questionnaire focused on the sensory characteristics of taste and texture to measure the reaction for four types of burger patties.

Participants were asked to state their age, gender, and diet on the questionnaire. Participants were then asked to taste pieces of vegan and vegetarian burger patties made from *A. oryzae* as well as Beyond and Quorn. The reason that Beyond and Quorn burger patties were included in the tasting was to provide participants with reference points when they rated the minimally processed fungi burger patties made from *A. oryzae.*

The sample size of each burger served to each participant was an eighth of a burger patty. The samples were served on one plate and were labelled so participants knew which burger corresponded to each section of the questionnaire. The participants were informed about the identity of each of the four samples verbally and in writing. The participants were permitted to taste as much of the sample as they wanted while indicating their liking of the taste and texture for each burger patty (Appendix A). While a 9-point hedonic scale is beneficial in assessing small differences across samples (Lawless & Heymann, 2013), a 5-point scale (1 = Like extremely, 2 = like, 3 = neither like nor dislike, 4 = dislike, 5 = dislike extremely) was used in the current study because of the major differences across the four burger patty samples [48] (e.g., in terms of ingredients, seasoning and production). 

The participants were also encouraged to notate any further remarks for each respective burger patty. In the section that focused on the taste of the burgers, participants were encouraged to share additional comments through the provision of trigger questions such as ‘how did you like aspects such as salty, savory, sweet, sour and umami flavors?’ and ‘how could the taste be improved?’. Likewise, the participants were encouraged to share further comments in the section that focused on the texture through trigger questions including ‘was chewiness or juiciness okay?’, ‘was it hard or dry?’, ‘did it resemble meat?’ and ‘how could the texture be improved?’ (Appendix A).

#### 2.2.4. Data Analysis

To satisfy the objective of assessing how participants evaluated the taste and texture of food made from minimally processed *A. oryzae* biomass, the data were analyzed and presented descriptively. The way in which the minimally processed biomass was reviewed against the established and commercially processed products was also descriptively assessed. A summative content analysis was performed on qualitative data to review the comments of the participants shared in detail and to identify key messages [49]. Participants’ ratings regarding the taste and texture of the four samples were analyzed collectively to assess the findings of the sensory experience.

Despite the relatively small number of participants (*n* = 15) able to participate in the tasting due to COVID-19 restrictions, a statistical analysis comprising of cross-tabulation, the Fisher Exact test and Cramer’s V-test performed using IBM^®^ SPSS (version 27, USA) was performed. This was performed to obtain tentative data that indicates whether or not there may be statistical relations of age and gender with preference profiles regarding the reception of the samples’ characteristics. The statistical importance between the independent variables (age and gender) and the outcome variable (liking of taste and texture of the burger patties) at a significance level of 5% were determined using the Fisher Exact test rather than a Chi-square analysis for association because few observations were expected for individual cells [50].

## 3. Results and Discussion

This study explored the suitability of a minimally processed burger patty derived from an *A. oryzae* biomass grown via SmF without an external pH adjustment. The production began with the propagation of fungal spores which were then used to prepare a preculture in 1 L shake flasks. The preculture was used as seeding for a 26 L reactor and the biomass from the 26 L reactor was used to inoculate the 1200 L air lift reactor. Oat flour was the only carbon and nitrogen source for the cultivation of *A. oryzae,* without any form of supplementation in the pilot bioreactor. A suitable substrate concentration for the fermentation process was first determined in shake flask trials, and the scaling up was performed stepwise. The harvested biomass from the pilot bioreactor was used to create vegan and vegetarian fungi burger patties with minimal downstream processing. The food products were then used for a sensory evaluation using an untrained panel. The data collected from the panel was finally analyzed to determine perceptions regarding the burgers’ taste and texture.

### 3.1. Substrate Preparation

#### 3.1.1. Defining Appropriate Substrate Concentration According to Biomass Yield and Residual Starch in the Medium

**Figure 6:**
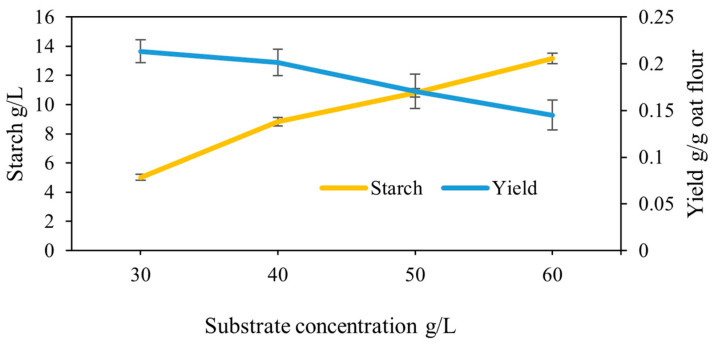
Profile of biomass yield, substrate concentration and starch consumption during growth of *Aspergillus oryzae* on oat flour in shake flasks.

To assess the appropriate substrate concentration for most constructively influencing the biomass yield in large scale productions, four concentrations of oat flour 30, 40, 50 and 60 g/L were used for fungal growth in shake flasks. The fungi started to grow after 6 h, and no enzymatic supplementation was required. The biomass was harvested after 48 h. The results showed that with an increasing substrate concentration from 30 to 60 g/L, the biomass yield decreased from 0.21 to 0.14 g/g oat flour (Figure 6). However, there were no statistically significant differences in the yield of the biomass (*p* = 0.105). The relation of the lowered yield value with the increased substrate concentration was previously also reported for the submerged cultivation of *Rhizopus delemar* on bread [51].

An increasing substrate concentration was found not to benefit the consumption of starch by the fungus during the 48 h of cultivation. As the substrate concentration increased from 30 g/L to 60 g/L, the starch concentration in the medium rose from 4 to 13 g/L (Figure 1). In starch enzymatic hydrolysis processes, inhibitory effects may occur during the liquefaction or saccharification stages due to the presence of high concentrations of glucose or starch. These inhibitory effects can cause an undesirable decrease in the enzyme’s catalytic activity. Previous research showed that at high starch concentrations, a reduction in enzymatic activity can occur during starch hydrolysis, and that glucose acts as a competitive inhibitor of the process [52,53,54]. Given this information, the 30 g/L oat concentration was scaled up for large-scale fermentation.

#### 3.1.2. Defining Appropriate Water Temperature for Achieving Ideal Mixing Condition

After the suitable substrate concentration was identified, it was necessary to examine how the media viscosity could be decreased for easy transferal of the 30 g/L oat flour concentration into the 1000 L airlift reactor. The result showed that increasing the temperature from 50 to 90 °C was aligned with increased media viscosity. The viscosity of the media with cold water (22 °C) and 50 °C water was more proper for easy transferring. This issue can be explained because of starch gelatinization during heating in excess water which causes swelling and disruption when the semi-crystalline structure melts. To avoid this, cold water (22 °C) was used for media preparation as it was easy to transfer and cost-effective because no extra energy was needed for heating.

### 3.2. Submerged Fermentation in the Pilot Bioreactor

#### Monitoring Biomass Weight, pH, and Starch Concentration during Fermentation Process

**Figure 7:**
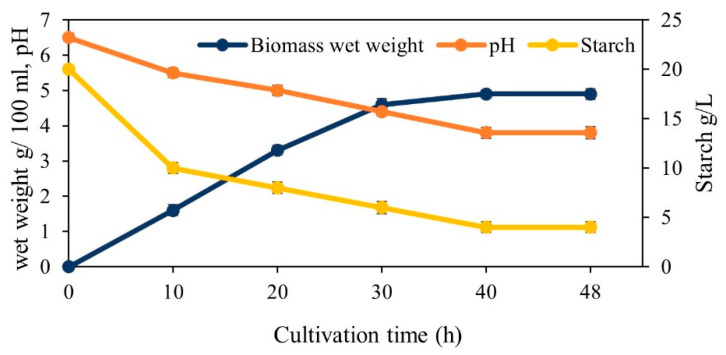
Monitoring the biomass wet weight, pH and starch consumption during growth of *Aspergillus oryzae* on oat flour in a 1200 L airlift reactor.

The monitoring of the fermentation process is critical, especially in large-scale fermentation, to control productivity and obtain high product quality. Monitoring can represent the chemical, physical and biological status of the culture and help to realize and correct any deviations from the specified optimum conditions and to also determine the ideal time to harvest [55]. In this study, the wet weight of biomass and the medium pH were used as indicators of fungal growth. After 10 h of cultivation, during which the initial pH was decreased from 6.5 to 5.5, 1.6 g of the wet biomass/100 mL of the removed culture was measured. After 20 h, the pH continued to decrease to 5, and the biomass weight roughly doubled from 1.6 g/100 mL to 3.3 g/100 mL. After 30 h, the pH further reduced to 4.4, and the biomass increased to 4.6 g/100 mL. After 40 h, the pH decreased to 3.8, and the biomass weight increased by just 0.3 g (4.9 g/100 mL). No further increase in the biomass weight was observed between hour 40 and 48. The biomass was thus harvested after 48 h of cultivation, given that the fermentation process appeared to have reached a stationary phase (Figure 7). The biomass concentration at the end of cultivation was 6 g/L at a yield of 0.2 g/g oat flour which was similar to the outcome of the shake flask experiment. 

During the cultivation, the starch concentration of the culture was also monitored to assess the amount of substrate accessible for hydrolysis. The initial starch content of 20 g/L (around 70% of oat composition) decreased throughout the duration of cultivation to 4 g/L upon harvest. Within the first 10 h of cultivation, 50% of starch was hydrolyzed while the pH ranged between 5.5 to 6.5. Such fast starch hydrolysis is in agreement with the optimum pH (6) for amylase activity of *A. oryzae* [56]. Previous research found that lowering the pH from 6 to 3 decreased the amylase activity of *A. oryzae* from 16.99 to 7.17 Iu/mL [56]. This was mirrored in the finding of the current study, as decreasing the pH drastically reduced the fungus’ ability to hydrolyze starch. While 10 g/L starch was broken down within 10 h when the pH ranged from 5.5 to 6.5, only 6 g/L starch was hydrolyzed when the pH dropped to 3.8–5.5 during the remaining 38 h of cultivation. The pH remained constant at 3.8 during the last 8 h of cultivation, and no starch hydrolysis was observed during this period. Upon harvest, 4 g/L of the oat flour remained in the culture. Therefore, the enzyme activity was markedly affected by pH [57]. Use of acidic pH has been reported as the most efficient method for amylase deactivation [52]. In addition to the pH, a high concentration of glucose or starch in the media can also decrease enzyme activity due to inhibitory effects.

### 3.3. Protein Content of Fungal Biomass

During 48 h of cultivation, the nutritional value in terms of protein content increased from 11% in the oat flour to 37% in the unwashed fungal biomass (on a dry weight basis). A similar increase was also observed in a study on the SmF of *Rhizopus delemar* on bread, in which the protein content increased from 13% to 30% [51]. The protein content of the *A. oryzae*-based biomass found in the current study also roughly compares with commercially available fungi-based products by Quorn which contain up to 44% protein [58]. 

Protein, however, is only one among other essential macronutrients important in food products. Fungal fermentation not only improves the profile of macronutrients, such as protein, but also that of micronutrients including dietary fiber, essential amino acids, vitamins, and minerals [12,58]. Yet, the nutritional profile of fungi-based products is expected to vary based on the substrate, the fungus cultivated, the way the biomass is further processed into food products as well as the presence of other ingredients in final products. To establish the nutritional profile of *A. oryzae* cultivated on oat flour in SmF, it may be beneficial to analyze both the derived biomass as well as products made from the ingredient in future.

The hyphae of the fungus tangled during cultivation, and this resulted in clumps of mycelium. The harvested fungal biomass was used to make fungal burger patties because these clumps with interwoven fungal mycelia are useful to further enhance the texture of the food products by introducing, e.g., more chewiness [59]. The biomass was dewatered prior to processing into vegan and vegetarian burger patties.

### 3.4. Sensory Evaluation 

The burger patties were rated by an untrained panel. A total of fifteen (*n* = 15) questionnaires were analyzed. Among the participants were seven women and eight men ranging between 27 and 62 years of age (Table 1), none of whom were vegan or vegetarian.

#### 3.4.1. Taste 

The collective rating profiles regarding taste showed that participants mostly rated that they neither liked nor disliked or disliked the taste of the vegan fungi burger (Table 2). Participants remarked that the vegan fungi burger tasted slightly salty, bitter and sour. The findings indicate that very little salt is needed in the production of *A. oryzae*-based foods. This is likely due to the fungus’ inherent umami characteristic [21]. The fungus’ taste also differs depending on the nutritional values of the substrate used for its growth. For example, koji, which is rice fermented with *A. oryzae* in solid state fermentation, tends to taste sweeter than the *A. oryzae* fermented grain does because rice is richer in starches [21]. The taste of *A. oryzae* also depends on chemical compounds and enzymes which are released when the hyphen of the fungus penetrate the substrate and absorb the substrate’s nutrition to enhance the fungus’ metabolism [21]. This is because these enzymes break starch, protein and fats down into their individual components [60]. Yet, taste aspects can also be redesigned when products containing *A. oryzae* are further treated through, e.g., specific heat treatments [61]. Because this study aimed to assess the use of *A. oryzae* after minimal processing, this process was not performed.

Some of the participants rated ‘extremely’ regarding the extent to which they liked the taste of the vegetarian fungi burger. Half of the participants rated that they either liked or neither liked nor disliked it. Some participants also rated that they disliked or extremely disliked the vegetarian burger’s taste. Participants remarked on similar issues as they had in the case of the vegan fungi-burger. Some found the vegetarian fungi burger too salty, bitter and sour. A few participants also remarked that it tasted stale. Yet, others remarked on its umami properties. Because taste characteristics can be altered or masked with, for example, spices, the results indicate that the way the biomass from *A. oryzae* is seasoned is important. Yet, the fungus was minimally processed and minimally spiced for the purpose of this study. Future research should target assessing the most favorable flavors to combine this fungus with as well as the most suitable substrate to grow *A. oryzae* on to achieve a biomass that does not require extensive treatment and alterations after cultivation. 

The majority of the participants rated that they extremely liked or liked the Beyond burger patty. Some participants remarked that the Beyond burger’s taste was lighter than that of the two *A. oryzae*-based burgers. Furthermore, some participants pointed out that its taste was similar to that of meat, while others remarked that they did not think that it tasted like meat. Most of the participants rated that they either extremely liked or liked the taste of the Quorn burger. A quarter of the participants rated that they either neither liked nor disliked this burger or that they disliked it. These findings are interesting in light of a tasting, which found that roughly the same number of participants preferred a fungi-based burger (*Neurospora intermedia*-based) over a Quorn one (*Fusarium venenatum-*based) and vice versa [62]. The participants of the current study provided that they like the taste of this burger too but that they preferred the Beyond burger’s taste. Some participants remarked that the saltiness should be adjusted but did not evolve in which way. Some participants remarked that they enjoyed the grilled taste of the Quorn burger. 

#### 3.4.2. Texture 

Roughly half of the participants neither liked nor disliked the texture of the vegan fungi burger while many disliked its texture (Table 3). Some of the participants remarked that the texture was okay for them, and others stated that the texture was too soft and smooth, and that they desired juiciness and a harder texture. One participant suggested that the texture could be improved through cooking. This is, in fact, true, and can be accomplished by drying the biomass out more thoroughly post fermentation. Moreover, the texture of submerged fermented *A. oryzae* can also be industrially improved through texturizing in similar ways as is done in the case of textured vegetable protein. 

Alternatively, future research might assess whether SSF of *A. oryzae* improves the response to the texture of food made from the fungus. It is worth mentioning that SmF, performed in the current study, can achieve a final biomass that contains less substrate than biomass from SSF since the former consists of more dissolved nutrients than the latter. It is expected that SSF would provide a more favorable texture. This is because the results of another tasting showed that the majority of participants liked the texture of fungal-burger patties made through solid-state fermentation [62]. In solid-state fermentation, hyphae bridge the gaps between pieces of substrate, and thereby form an interconnected sheet held together by the mycelium of the fungus [12,21]. Further research on texture improvements of food made from *A. oryzae* would be especially interesting given the novelty of using the fungus in the production of food products as opposed to using the biomass for secondary fermentation such as soy sauce production. 

One third of the participants neither liked nor disliked the texture of the vegetarian fungi burger. While some participants extremely liked or liked the texture of the vegetarian burger, many disliked or extremely disliked its texture. Some participants pointed out that they disliked the softness and wished for more dryness, a harder texture and more chewiness. Some also noted that the vegetarian fungi burger did not resemble meat. Some participants highlighted that they enjoyed the melt-in-the-mouth sensation of the vegetarian fungi burger. The use of egg white in the vegetarian burger seems to have made a significant difference on the extent to which participants liked the texture of this burger. Future research may assess whether powdered egg whites would be a more suitable ingredient given that many participants remarked that they thought that both fungi burgers were slightly too soft and smooth. Yet, future research may also assess how the use of other binders or ingredients are able to improve the texture of products made from *A. oryzae*.

Most of the participants liked the Beyond burger’s texture, and many extremely liked its texture. Some neither liked or disliked it and few disliked or extremely disliked the Beyond burger’s texture. Some participants thought the Beyond burger’s juiciness and chewiness were just right and that they perceived it to be meat-like. Roughly one half of the participants liked the Quorn burger’s texture whereas a quarter each rated that they liked this burger’s texture or that they neither liked nor disliked it. The findings of the current study are interesting in light of a study that found that roughly a quarter of participants preferred the texture of burger patties made from *Neurospora intermedia* whereas a third of participants preferred the texture of Quorn burger patties [62]. While many of the current study’s participants’ comments mirrored those shared about the Beyond burger’s texture, some participants found the texture of the Quorn burger too dry. Other participants shared that they thought that the textures of the Quorn burger and the vegan fungi burger were very similar.

Overall, future research may assess how the texture of patties made from *A. oryzae* biomass can be further improved. There are many possible methods through which to improve texture, including the strategies employed by Quorn. The role of *A. oryzae* as a source for other food products should also be further considered. This may be especially interesting when the biomass is used alternatively to products that are expected to have a soft texture, such as tofu. Given that none of the participants in the current study were vegetarians or vegans, further studies may also assess how those who follow such diets respond to food made from *A. oryzae* biomass. This is because non-vegan and non-vegetarians who taste meat analogues tend to rate them lower because they inadvertently expect sensory characteristics to resemble those of meat [63].

#### 3.4.3. Statistical Relations

Two statistical relations were found. Age was statistically related to the liking of the taste of the vegan fungi burger (*p* = 0.019) at a significance level of 5% and 2.5% (*p* > 0.025). Participants over the age of 35 years tended to neither like nor dislike or like the vegan fungi burger whereas younger participants tended to dislike or extremely dislike the vegan fungi burger. Age was statistically related to the reaction to the texture of the vegetarian fungi burger at a significance level of 5% (*p* = 0.039). The majority of participants under the age of 35 years either disliked or extremely disliked this characteristic whereas the majority of participants over the age of 35 years neither liked nor disliked the texture of the vegetarian fungi burger. This finding is not in agreement with research that found that neither age nor gender were statistically associated with the liking of taste and texture of another fungi-based burger patty for which *Neurospora intermedia* was cultivated on bread in SSF [62]. Yet, the number of participants was relatively small (*n* = 15) in the current study, which is why future research with larger numbers of participants is needed to assess statistical relations across age, gender and other socio-demographics in order to draw constructive conclusions.

### 3.5. Limitation of the Study

The current study assessed the submerged cultivation of *A. oryzae* on oats in pilot scale without specific modifications. Yet, the starch conversion and thus biomass yield could have been higher if pH, C/N ratio, and vitamin and mineral supplementation would have been adapted during the course of the cultivation of the fungus. In terms of the results of the sensory analysis, the number of participants was relatively small due to the COVID pandemic. The sample size was sufficient in providing a general idea of how participants perceived minimally processed food made from *A. oryzae*. Yet, the results and conclusions derived from this study may not be representative on a national level.

## 4. Conclusions

*A. oryzae* has the potential to grow under submerged fermentation and solely on oat flour without the addition of supplements to assist its growth. The fungus produces nutritious protein-rich biomass that can be converted to various food products that are functional in nature. The results indicate the development of promising applications for the use of *A. oryzae* in food production. Yet, the most favorable ways to texture and season this relatively new food source to the preference of consumers needs to be further explored. This is particularly the case in cases where *A. oryzae*-based biomass is to be used to make food products as opposed to be used for secondary fermentation as it has commonly been.

## Figures and Tables

**Table 1:** Participant profile.

Gender	Age Group 1 (27–34)	Age Group 2 (35–62)
Female	20%	26%
Male	27%	26%
Combined	47%	53%

**Table 2:** Preference profiles regarding taste across the participants (*n* = 15).

	Vegan	Vegetarian	Beyond	Quorn
1—Like extremely	0	2	5	4
2—Like	2	4	6	7
3—Neither like nor dislike	6	4	2	3
4—Dislike	4	2	1	1
5—Dislike extremely	3	3	1	0

**Table 3:** Preference profiles regarding texture across the participants (*n* = 15).

	Vegan	Vegetarian	Beyond	Quorn
1—Like extremely	0	1	4	4
2—Like	1	1	7	7
3—Neither like nor dislike	7	5	2	4
4—Dislike	6	3	1	0
5—Dislike extremely	1	5	1	0

**Figure A1:**
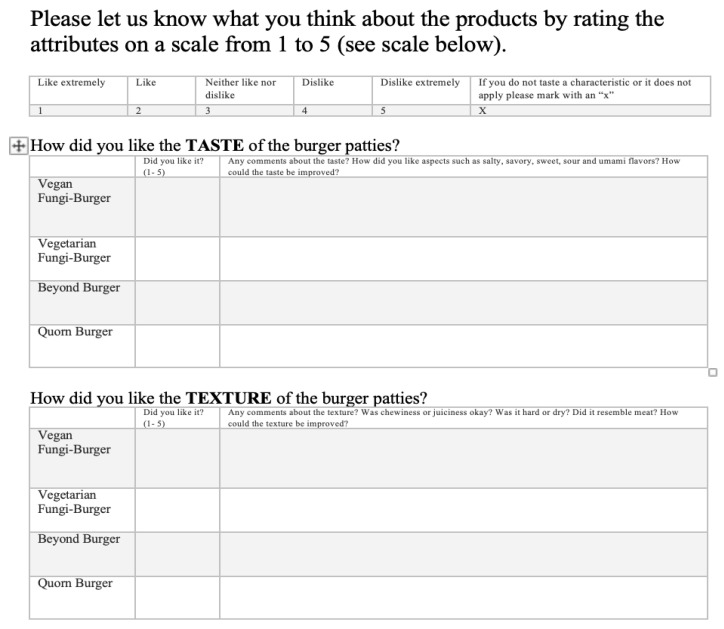
Questionnaire used in the sensory evaluation.
